# Effect of Central Administration of Brain-Derived Neurotrophic Factor (BDNF) on Behavior and Brain Monoamine Metabolism in New Recombinant Mouse Lines Differing by 5-HT_1A_ Receptor Functionality

**DOI:** 10.3390/ijms222111987

**Published:** 2021-11-05

**Authors:** Darya Bazovkina, Vladimir Naumenko, Ekaterina Bazhenova, Elena Kondaurova

**Affiliations:** Institute of Cytology and Genetics, Siberian Division of the Russian Academy of Science, 630090 Novosibirsk, Russia; naumenko2002@mail.ru (V.N.); ekaterina.yu.bazhenova@gmail.com (E.B.); kond_em@bionet.nsc.ru (E.K.)

**Keywords:** BDNF, aggression, 5-HT_1A_ and 5-HT_2A_ receptors functional activity, brain serotonin system, brain dopamine system, HPLC, recombinant mouse lines

## Abstract

Experiments were carried out on recombinant B6.CBA-D13Mit76C (B6-M76C) and B6.CBA-D13Mit76B (B6-M76B) mouse lines created by transferring a 102.73–118.83 Mbp fragment of chromosome 13, containing the 5-HT_1A_ receptor gene, from CBA or C57BL/6 strains to a C57BL/6 genetic background, correspondingly. We have recently shown different levels of 5-HT1A receptor functionality in these mouse lines. The administration of BDNF (300 ng/mouse, i.c.v.) increased the levels of exploratory activity and intermale aggression only in B6-M76B mice, without affecting depressive-like behavior in both lines. In B6-M76B mice the behavioral alterations were accompanied by a decrease in the 5-HT_2A_ receptor functional activity and the augmentation of levels of serotonin and its main metabolite, 5-HIAA (5-hydroxyindoleacetic acid), in the midbrain. Moreover, the levels of dopamine and its main metabolites, HVA (homovanillic acid) and DOPAC (3,4-dihydroxyphenylacetic acid), were also elevated in the striatum of B6-M76B mice after BDNF treatment. In B6-M76C mice, central BDNF administration led only to a reduction in the functional activity of the 5-HT_1A_ receptor and a rise in DOPAC levels in the midbrain. The obtained data suggest the importance of the 102.73–118.83 Mbp fragment of mouse chromosome 13, which contains the 5-HT_1A_ receptor gene, for BDNF-induced alterations in behavior and the brain monoamine system.

## 1. Introduction

Brain-derived neurotrophic factor (BDNF) is known to be involved in the pathogenesis of many disorders of the nervous system, including aggression and depression [1,2,3,4,5,6]. This neurotrophic factor is a promising drug for therapeutic applications [7,8].

There are many data indicating the cross-talk interaction between the brain monoamine system and BDNF. For example, central BDNF administration increases the level of serotonin (5-HT) and its metabolite, 5-HIAA, in adult rat brain structures [9]. The conditional BDNF knockout results in a significant reduction in 5-HT_2A_ receptor density in the frontal cortex and 5-HT_1A_ receptor density in the hippocampus, with corresponding changes in the gene expression of the 5-HT_2A_ and 5-HT_1A_ receptors [10]. On the other hand, there are plenty of data demonstrating an increase in brain BDNF levels after chronic treatment with classical antidepressants—selective serotonin reuptake inhibitors (SSRIs) [11,12,13]. In other words, BDNF can modulate the 5-HT system and vice versa.

Additionally, numerous lines of evidence indicate that BDNF interacts with the brain dopaminergic (DA) system. For example, exogenous BDNF was shown to promote the survival and differentiation of cultured dopaminergic neurons [14,15]. Additionally, striatal in vivo infusions of BDNF locally elevated the release of DA [16]. On the other hand, the increased expression of BDNF protein in the cortex and hippocampus after the subchronic administration of rotigotine, a DA agonist of the non-ergoline class, was observed [17].

It is noteworthy that 5-HT/DA interactions also exist and play an important role in the regulation of behavior and the pathophysiology of several neuropsychiatric disorders, including depression, aggression and Parkinson’s as well as Alzheimer’s diseases [18,19]. Although many data, including our own previous results [20,21], strongly suggest the involvement of the brain 5-HT and DA systems in the behavioral effects of BDNF, there is still a lack of data on the effect of the genotype on the BDNF action on behavior and the brain 5-HT and DA systems.

Recombinant mouse lines differed only by a small genome fragment, representing interesting and promising models for studying the influence of genetic modification on the molecular mechanisms of behavior and drug responses. Earlier we studied the effect of the single i.c.v. BDNF administration on behavior and the brain 5-HT system in the AKR.CBA-D13Mit76C mouse line obtained by transferring a distal fragment of chromosome 13, containing the 5-HT_1A_ receptor gene, from a CBA/Lac strain to an AKR/J genetic background. BDNF treatment restored social deficiency in AKR.CBA-D13Mit76C mice but produced asocial behavior (increased duration of aggressive attacks towards juvenile mice) in animals of a parental AKR strain. These effects of BDNF were accompanied by decreased functional activity of the 5-HT_2A_ receptor in AKR.CBA-D13Mit76C mice and increased functional activity of it in AKR mice [20].

However, the results obtained with the AKR.CBA-D13Mit76 line are difficult to compare with other animal models, since the AKR genotype is not generally utilized as a genetic background for transgenic mice. In order to solve this problem we transferred a CBA-derived 102.73–110.56 Mbp fragment of chromosome 13 from a CBA/Lac strain to the genome of C57BL/6 mice. As a result, we created the B6.CBA-D13Mit76C (B6-M76C) and B6.CBA-D13Mit76B (B6-M76B) recombinant lines with the genetic background of C57BL/6, distinguished by the CBA- or C57BL/6-derived fragment of chromosome 13 containing the 5-HT_1A_ receptor gene. Recently, we suggested that B6-M76C mice demonstrated increased postsynaptic and decreased pre-synaptic functional responses of the 5-HT_1A_ receptor in comparison with B6-M76B animals [22]. Moreover, we revealed the significant changes in the effects of fluoxetine treatment on the behavior of and brain 5-HT system in B6-M76C mice. In contrast to B6-M76B mice, in B6-M76C mice fluoxetine produced a pro-depressive effect, assessed in a forced swim test [23], which confirmed our idea on altered 5-HT_1A_ receptor functionality in B6-M76C mice.

There is some evidence indicating the role of presynaptic 5-HT_1A_ receptors in the regulation of aggression [24] and the mechanism of antidepressant drug action [20,25,26]. An increased density of presynaptic 5-HT_1A_ receptors was observed in depressed patients and suicidal victims [27,28]. Preclinical data also show that the activation of postsynaptic 5-HT_1A_ receptors is important for the antidepressant effect of 5-HT_1A_ receptor agonists [29]. In view of the above, we suppose that BDNF exposure could produce different influences on the behavior and brain monoamine system of recombinant B6-M76B and B6-M76C mice.

In the current paper, in order to further investigate the link between BDNF and the brain 5-HT and DA systems, we compared the chronic effect of central, single administration of BDNF on: (1) locomotor and exploratory activity, anxiety, depressive-like behavior and aggression; (2) functional activities of 5-HT_1A_ and 5-HT_2A_ receptors; and (3) levels of major neurotransmitters, namely dopamine and serotonin, along with their main metabolites, homovanillic acid (HVA), 3,4-dihydroxyphenylacetic acid (DOPAC) and 5-hydroxyindoleacetic acid (5-HIAA), in the brain structures of B6-M76B and B6-M76C recombinant mouse lines differentiated by 5-HT_1A_ receptor functionality.

## 2. Results

### 2.1. Behavioral Studies

**Figure 1:**
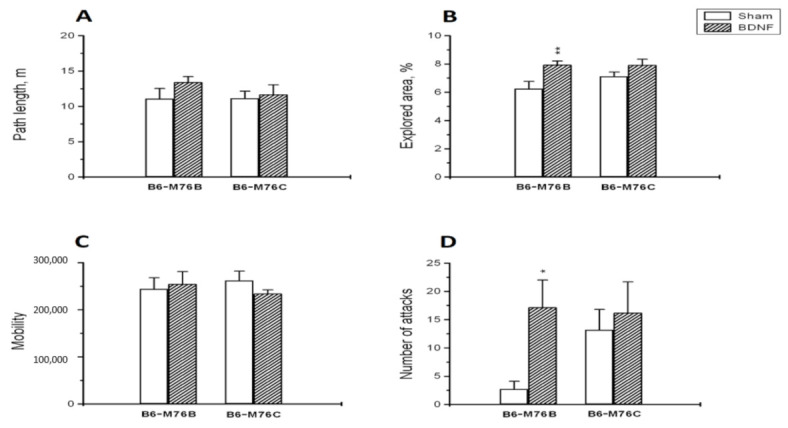
The effect of intracerebroventricular BDNF administration on the behavior of B6-M76B and B6-M76C mice. The influence of BDNF on the (**A**) path length, (**B**) explored area in the open-field test, (**C**) mobility in the forced swim test and (**D**) number of aggressive attacks in the resident–intruder test is shown. All magnitudes are presented as the mean ± SEM of at least 7 animals per group, and compared using two-way ANOVA. *, *p* < 0.05 and **, *p* < 0.01 vs. the sham group of the same line.

BDNF administration significantly increased (*p* < 0.01) the percent of the explored area of the open field in B6-M76B but not in B6-M76C mice (Figure 1B): two-way ANOVA showed the effect of the treatment (F1.29 = 9.9, *p* < 0.01), but it revealed no effect of the genotype (F1.29 = 1.1, *p* > 0.05) or the treatment x genotype interaction (F1.29 = 1.3, *p* > 0.05). However, BDNF failed to alter all other behavioral traits estimated in the open-field test. Two-way ANOVA revealed no effect of the treatment (F1.29 = 0.1, *p* > 0.05), genotype (F1.29 = 0.3, *p* > 0.05) or the treatment x genotype interaction (F1.29 = 2.0, *p* > 0.05) for traveled path length (Figure 1A), as well as for time spent in center (F1.29 = 0.4, *p* > 0.05 for the treatment, F1.29 = 1.3, >0.05 for the genotype and F1.29 = 0.5, *p* > 0.05 for the treatment x genotype interaction). For the B6-M76B line, the time spent in center: control group—4.5 ± 0.6%, BDNF group—7.6 ± 1.2%. For the B6-M76C line, the time spent in center: control group—7.1 ± 1.9%, BDNF group—5.8 ± 1.0%.

It is noteworthy that BDNF failed to alter the mobility level in the forced swim test (Figure 1C): two-way ANOVA showed no effect for the treatment (F1.29 = 0.004, *p* > 0.05), the genotype (F1.29 = 0.1, *p* > 0.05) or the treatment x genotype interaction (F1.29 = 0.8, *p* > 0.05).

B6-M76B mice treated with BDNF showed a higher number of attacks in the resident–intruder test compared with mice from the sham group (*p* < 0.05) (Figure 1D). Meanwhile, two-way ANOVA revealed only an effect of the treatment (F1.29 = 3.9, *p* < 0.05), but not for the genotype (F1.29 = 1.2, *p* > 0.05) or the treatment x genotype interaction (F1.29 = 1.6, *p* > 0.05). It is noteworthy that B6-M76B mice of the sham group demonstrated fewer instances of attack than B6-M76C mice from the sham group; however, this difference was below the significance threshold (*p* = 0.08) (Figure 1D). At the same time, BDNF did not affect the duration of time of attacks: two-way ANOVA showed no effect for the treatment (F1.29 = 0.5, *p* > 0.05), the genotype (F1.29 = 2.0, *p* > 0.05) or the treatment x genotype interaction (F1.29 = 0.4, *p* > 0.05). The duration of time of attacks was, for the B6-M76B line: control group—13.0 ± 8.4 s, BDNF group—41.2 ± 14.4 s. For the B6-M76C line: control group—31.7 ± 10.2 s, BDNF group—41.8 ± 17.0 s.

### 2.2. 5-HT_1A_ and 5-HT_2A_ Receptors Functional Responses

**Figure 2:**
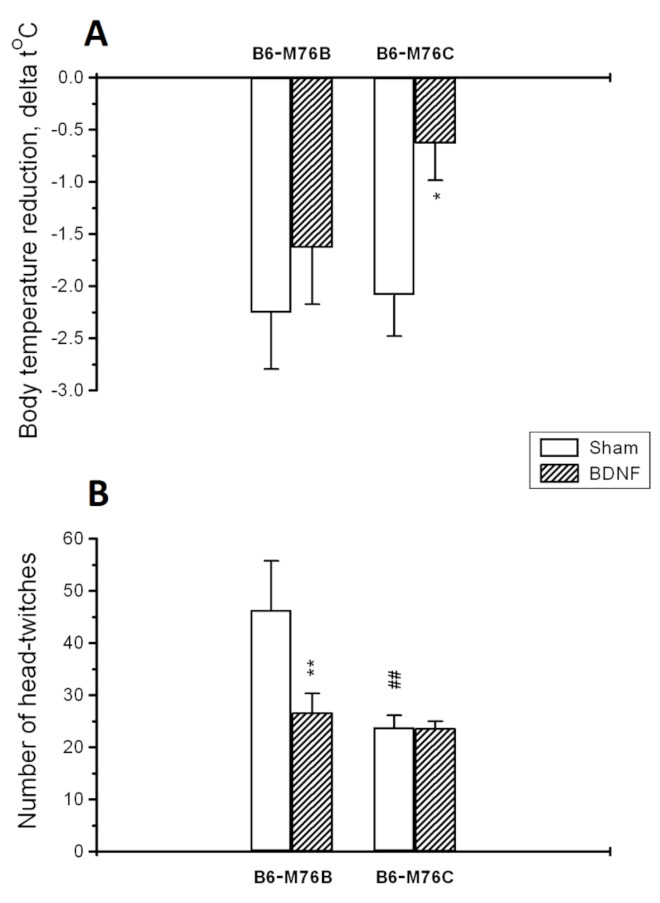
Functional activities of 5-HT1A (**A**) and 5-HT2A receptors (**B**) in the brain of B6-M76B and B6-M76C mice treated with BDNF as well as the sham groups. The functional response of 5-HT1A receptor was estimated by intensity of a 5-HT1A-receptor-mediated hypothermic reaction induced by the acute administration of 8-OH-DPAT (1 mg/kg, i.p.). The functional response of the 5-HT_2A_ receptor was estimated by the number of 5-HT_2A_receptor-mediated head twitches induced by the acute administration of DOI (1 mg/kg, i.p.). All magnitudes are presented as the mean ± SEM of at least 7 animals per group and compared using two-way ANOVA. *, *p* < 0.05 and **, *p* < 0.01 vs. the sham group of the same line; ^##^, *p* < 0.01 vs. the B6-M76B sham group.

BDNF significantly changed the functional activities of both 5-HT_1A_ and 5-HT_2A_ receptors in different ways (Figure 2A,B). A 5-HT_1A_-receptor-induced hypothermic response was reduced only in B6-M76C mice after BDNF treatment (*p* < 0.05) (Figure 2A). Two-way ANOVA showed an effect of the treatment (F1.28 = 5.3, *p* < 0.05), but there were no effects of the genotype (F1.28 = 1.7, *p* > 0.05) or the treatment x genotype interaction (F1.28 = 0.9, *p* > 0.05) on the 5-HT_1A_ receptor functional activity.

The number of 5-HT_2A_receptor-induced head twitches was reduced only in B6-M76B mice after BDNF exposure (*p* < 0.01) (Figure 2B). Two-way ANOVA revealed the effects of the treatment (F1.29 = 4.5, *p* < 0.05), the genotype (F1.29 = 7.6, *p* < 0.01) and the treatment x genotype interaction (F1.29 = 4.5, *p* < 0.05) on the 5-HT_2A_ receptor functional response. It is noteworthy that the number of 5-HT_2A_receptor-induced head twitches was lower in B6-M76C mice of the sham group compared with sham B6-M76B mice (*p* < 0.01), indicating reduced 5-HT2A receptor functional activity in B6-M76C mice (Figure 2B).

### 2.3. HPLC Analysis

**Figure 3:**
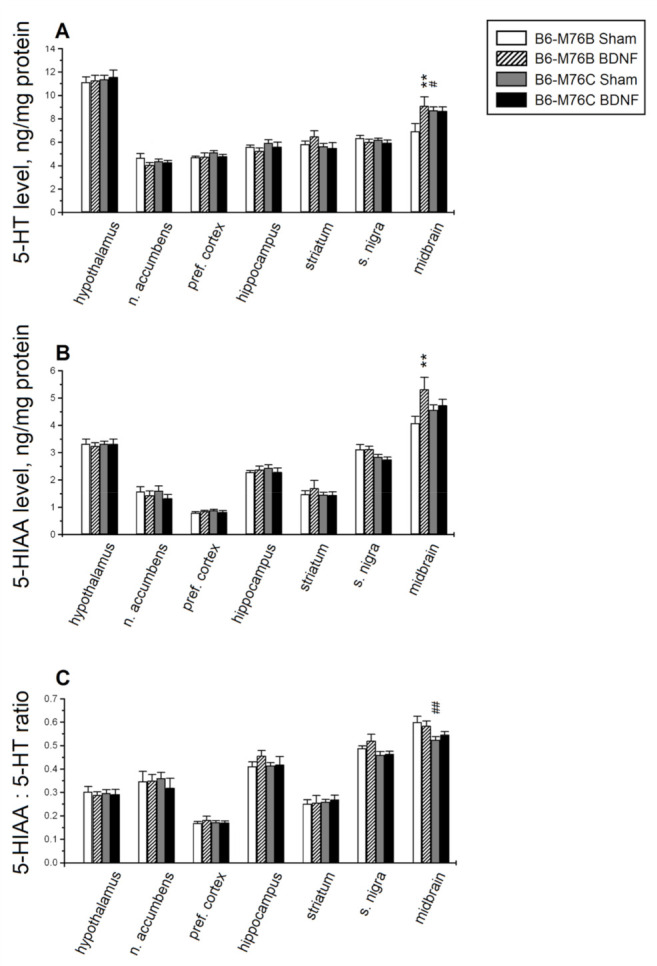
The effect of intracerebroventricular BDNF administration on concentrations of (**A**) serotonin (5-HT), (**B**) its main metabolite, 5-hydroxyindoleacetic acid (5-HIAA), and (**C**) the 5-HIAA/5-HT ratio (reflecting the efficiency of serotonin metabolism) in the different brain areas of B6-M76B and B6-M76C mice. All magnitudes are presented as the mean ± SEM of at least 7 animals per group and compared using two-way ANOVA. **, *p* < 0.01 vs. the sham group of the same line; ^#^, *p* < 0.05 and ^##^, *p* < 0.01 vs. the B6-M76B sham group.

Major differences in the serotonin level and its metabolism were found in the midbrain raphe nuclei area—the main brain region for the localization of 5-HT cell bodies. Two-way analysis of variance showed the effects of the treatment and the treatment x genotype interaction for 5-HT in the midbrain (Table 1). Post hoc analysis revealed that central BDNF administration resulted in the elevation (*p* < 0.01) of the 5-HT level in the midbrain of B6-M76B, but not B6-M76C, mice (Figure 3A). It should be noted that the 5-HT level in the midbrain of sham B6-M76C mice were higher compared to those for B6-M76B mice of the sham group (*p* < 0.05).

Two-way ANOVA showed, for the 5-HIAA level, the effect of treatment, as well as the tendency of the treatment x genotype interaction effect (*p* = 0.06) in the midbrain and the effect of the genotype in the substantia nigra (Table 1). Post hoc analysis demonstrated a rise in the 5-HIAA level in the midbrain of B6-M76B mice (*p* < 0.01, Figure 3B).

It is noteworthy that two-way ANOVA showed an effect of the genotype in the substantia nigra and in the midbrain for the 5-HIAA/5-HT ratio, reflecting 5-HT metabolism. However, Fisher’s post hoc analysis only revealed a difference (*p* < 0.01) in the midbrain 5-HIAA/5-HT ratio between B6-M76C and B6-M76B mice of the sham group (Figure 3C); this index was lower in B6-M76C mice.

**Figure 4:**
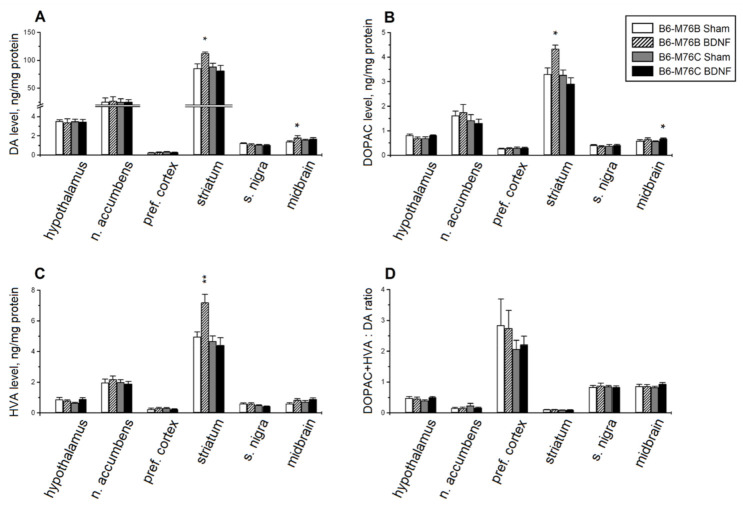
The effect of intracerebroventricular BDNF administration on concentrations of (**A**) dopamine (DA), its main metabolites, (**B**) 3,4-dihydroxyphenylacetic acid (DOPAC) and (**C**) homovanillic acid (HVA), and the (**D**) DOPAC+HVA/DA ratio (reflecting the efficiency of dopamine metabolism) in the different brain areas of B6-M76B and B6-M76C mice. All magnitudes are presented as the mean ± SEM of at least 7 animals per group and compared using two-way ANOVA. * *p* < 0.05, ** *p* < 0.01 vs. sham group of the same line.

BDNF significantly affected the brain levels of DA and its metabolites, DOPAC and HVA. Two-way ANOVA showed the effects of treatment in the midbrain and treatment x genotype interaction in the striatum for DA (Table 2). Post hoc analysis revealed a rise in the DA level in the striatum (*p* < 0.05) and in the midbrain (*p* < 0.05) of B6-M76B mice (Figure 4A). Two-way ANOVA detected the effects of the genotype and treatment x genotype interaction on DOPAC in the striatum and the effects of the genotype, treatment and treatment x genotype interaction on HVA in the striatum (Table 2). An increase in the levels of the DA metabolites, DOPAC (*p* < 0.05) and HVA (*p* < 0.01), was found in the striatum of B6-M76B mice (Figure 4B,C). Moreover, two-way ANOVA revealed the effects of the treatment on the midbrain and the treatment x genotype interaction on the hypothalamus for DOPAC, as well as the effects of the genotype on the substantia nigra and the treatment on the midbrain for HVA level (Table 2). However, post hoc analysis only demonstrated an increase in DOPAC levels in the midbrain of B6-M76C mice (*p* < 0.05) (Figure 4B).

Despite the observed alterations in the DA, DOPAC and HVA levels, BDNF failed to affect the DOPAC + HVA/DA ratio reflecting the DA metabolism (Figure 4D), although two-way ANOVA showed the effect of the genotype in the striatum (F1.28 = 5.20, *p* < 0.05).

## 3. Discussion

BDNF is important for neuronal survival during development and synaptic plasticity in adulthood. This is confirmed by the fact that most BDNF−/− mutants die during the second postnatal week [30]. It was shown that BDNF is involved in the regulation of different kinds of behavior, such as exploratory activity, anxiety, aggression [31,32] and depression-like behavior [30,33]; however, the precise mechanisms of BDNF action are not fully understood.

On the one hand, BDNF conditional knockout mice (with reduced BDNF levels in the forebrain) demonstrated increased levels of anxiety and aggression [32]. These mice also exhibited a depression-like phenotype in the tail suspension test, but not in the forced swim test [34]. However, BDNF+/− mice were indistinguishable from wild-type littermates in terms of the levels of locomotor and exploratory activities, anxiety and behavioral despair [35,36]. At the same time, BDNF+/− mice exhibited hyperaggression [36,37] and diminished responses to antidepressant treatment [38]. On the other hand, mice overexpressing BDNF in the forebrain, including the hippocampus and cortex, also displayed higher anxiety-like scores compared with wild-type littermate controls [31,39]. However, it should be noted that only hippocampal BDNF overexpression attenuated anxiety-like behaviors [40]. There is evidence that BDNF administration into the hippocampus produced an antidepressant effect in the standard behavioral models of depression—the learned helplessness and forced swim paradigms in rats [33]. Moreover, some authors showed that the mice overexpressing BDNF in the forebrain showed reduced immobility in the forced swim test [31]; a different study did not confirm this effect [39]. Thus, manipulations that increased or decreased the BDNF expression level do not always have a clear influence on depressive- or anxiety-like behavior, but these impaired BDNF expressions often lead to an increase in aggressive behavior.

This hypothesis is partially confirmed by our results: BDNF administration increased the level of intermale aggression and exploratory activity in the open-field test only in B6-M76B mice, without affecting the time spent in the center of the open-field arena or performance in the forced swim test in animals of both lines. Moreover, these results are consistent with previous ones: BDNF treatment induced asocial behavior towards juvenile mice in social interaction test only in AKR mice, and we did not reveal any BDNF effect on depressive- or anxiety-like behavior in mice of both AKR.CBA-D13Mit76C and AKR recombinant lines [20]. Thus, B6-M76C and AKR.CBA-D13Mit76C mice (carrying the CBA-derived distal fragment of chromosome 13 with the 5-HT_1A_ receptor gene on C57Bl/6 and AKR genetic backgrounds, correspondingly) are resistant to the “pro-aggressive” effect of BDNF central administration. These data indicate that the 102.73–118.83 Mbp fragment of chromosome 13 is important for aggressive behavior induced by BDNF exposure. It is noteworthy that the investigated fragment of chromosome 13 contains the 5-HT_1A_ receptor gene, which is known to be involved in the suppression of aggressive behavior [41,42,43,44]. Thus, it could be suggested that this receptor plays an important role in the regulation of BDNF-induced aggressive behavior.

Earlier, we found that the intensity of the hypothermic response evoked by a single 8-OH-DPAT injection in mice chronically treated with 8-OH-DPAT was significantly lower in B6-M76C than in B6-M76B animals. These results were in accordance with the data obtained from a cataleptic-prone CBA mouse strain [45], and it was suggested that in the B6-M76C line post-synaptic 5-HT_1A_ receptors undergo more effective desensitization [22]. Here, we found that central BDNF administration results in a reduction in 5-HT_1A_ receptor functionality in B6-M76C mice but not in B6-M76B mice. Taken together with the data on the central role of post-synaptic 5-HT_1A_ receptors in the mediation of 8-OH-DPAT-induced hypothermia [46,47], the current results confirm our previous suggestion.

In line with the fact that BDNF induced changes in the brain 5-HT system, it was shown that central BDNF administration results in an increase in 5-HT and 5-HIAA levels in the midbrain of B6-M76B, but not B6-M76C, mice. This in good agreement with the data indicating that central BDNF administration increases the levels of 5-HT and its metabolite, 5-HIAA, in adult rat brain structures [9]. It is necessary to note that all BDNF-induced changes in 5-HT and 5-HIAA levels were observed in the midbrain—a brain structure where the majority of 5-HT cell bodies as well as pre-synaptic 5-HT_1A_ receptors are localized [48]. At the same time, BDNF failed to alter the 5-HT brain metabolism in B6-M76C mice. However, our suggestion on the decreased functionality of the pre-synaptic 5-HT_1A_ receptor could explain this feature. The increased 5-HT level and reduced 5-HIAA/5-HT ratio in the midbrain of B6-M76C mice of the sham group also favor our suggestion on reduced 5-HT_1A_ autoreceptor activity, since these receptors are known to regulate the level of 5-HT in the synaptic cleft [48,49].

The effect of BDNF on 5-HT_2A_ receptor functionality is also of interest. BDNF administration led to a reduction in 5-HT_2A_ receptor-induced head-twitches in B6-M76B mice. This outcome is in agreement with the data of Trajkovska and co-authors, which showed that chronic BDNF exposure resulted in a specific decrease in 5-HT_2A_ receptor protein levels in hippocampal neuronal and slice cultures [50]. Earlier, we showed that BDNF increased 5-HT_2A_ receptor functionality in ASC mice genetically predisposed to depressive-like behavior [21], and also increased it in AKR but reduced it in AKR.CBA-D13Mit76C mice [20]. At the same time, in the present study we observed reduced 5-HT_2A_ functionality in sham B6-M76C mice. These data indicate the importance of the investigated fragment of chromosome 13 in the regulation of 5-HT_2A_ receptor functionality as well as in the response of the 5-HT_2A_ receptor to BDNF treatment. This is in the line with role of the 5-HT_1A_ receptor in the regulation of 5-HT_2A_ receptor functionality [47,51].

It should be acknowledged that BDNF caused considerable changes in the brain DA system in B6-M76B, but not B6-M76C, mice. Only a slight increase in DOPAC levels was revealed in the midbrain of B6-M76C, whereas a significant increase in DA, DOPAC and HVA levels in the striatum and DA levels in the midbrain of B6-M76B mice was shown. Since the nigrostriatal DA system is considered to be the center of sensomotor integration [52], the revealed BDNF-induced changes in the brain DA system may be the reason for the increase in exploratory activity in B6-M76B mice. On the other hand, it cannot be excluded that observed changes in the DA system are linked with the alterations in the 5-HT system, since these neurotransmitter systems are known to be closely related [53,54,55].

Thus, obtained data indicate the reduced reactivity of B6-M76C mice to BDNF, suggesting the importance of the 105.89–118.83 Mbp fragment of chromosome 13, containing the 5-HT_1A_ receptor gene, in the effect of BDNF on brain neurotransmitters, and hence on the behavior. Indeed, the present paper shows that the investigated fragment of chromosome 13 is essential for the development of BDNF-induced aggression. B6.CBA-D13Mit76C (B6-M76C) and B6.CBA-D13Mit76B (B6-M76B) recombinant mouse lines are distinguished by the CBA- or C57BL/6-derived fragment of chromosome 13 containing the 5-HT_1A_ receptor gene. We suppose that the 5-HT_1A_ receptor seems to be the main player in the realization of the discrepancies in response to BDNF. Firstly, we found that B6-M76C mice were characterized by an altered sensitivity of 5-HT_1A_ receptors to chronic 8-OH-DPAT (5-HT_1A_ receptor agonist) administration and higher 5-HT_1A_ receptor mRNA levels in the frontal cortex as well as the hippocampus in comparison to B6-M76B mice [22]. Moreover, we recently revealed significant changes in the effect of fluoxetine treatment on the behavior and brain 5-HT system of recombinant B6-M76C mice. In contrast to B6-M76B mice, fluoxetine produced a pro-depressive effect, assessed in a forced swim test in B6-M76C mice [23]. Consequently, the results of the current study confirmed our previous suggestion on the reduced functionality of the pre-synaptic 5-HT_1A_ receptors in B6-M76C mice.

## 4. Materials and Methods

### 4.1. Breeding of Lines

The creation of the recombinant B6.CBA-D13Mit76C (B6-M76C) and B6.CBA-D13Mit76B (B6-M76B) mouse lines by transferring the CBA-derived fragment 102.73–110.56 Mbp of chromosome 13 (including the 5-HT1A receptor gene at the position on 105.44 Mbp) to the C57Bl/6 (B6) genome has already been described [37]. Briefly, males generated in the Laboratory of Behavioral Neurogenomics (Novosibirsk, Russia) of the AKR.CBA-D13Mit76C recombinant line, containing the CBA-derived distal fragment of chromosome 13 in an AKR genetic background [56,57,58], and females of the B6 strain were mated to obtain the F1 hybrids. The B6-M76C and B6-M76B lines were produced by eight successive instances of backcrossing of the F1 hybrids to the B6 strain. The transfer of the CBA-derived fragment of chromosome 13 to the B6 genome was controlled using three polymorphic microsatellites: D13Mit287 (102.73 Mbp), D13Mit76 (110.56 Mbp) and D13Mit78 (118.83 Mbp). The heterozygous backcrosses of the eighth generation were intercrossed to generate B6-M76C and B6-M76B lines containing, respectively, the CBA- and B6-derived alleles of D13Mit287 and D13Mit76, as well as the AKR- and B6-derived alleles of D13Mit78 in the B6 genome. The equipment and mice maintenance were supported by the basic research project (0259-2021-0015).

### 4.2. Animals

Adult (10–12 weeks old) male mice of the B6-M76B (n = 7 for the sham group, n = 7 for the BDNF group) and B6-M76C (n = 10 for the sham group, n = 9 for the BDNF group) lines were used. Animals were housed in groups of 7–8 per cage, which were 40 cm × 25 cm × 15 cm, under standard conditions (20–22 °C, free access to food and water, 12 h light/dark cycle). Two days before behavioral testing, the mice were weighed (about 23 g) and isolated into individual cages to remove the group effect. All experimental procedures were in compliance with the EC Directive 86/609/EEC for animal experiments, and were approved by the Institute’s ethics committee. All efforts were made to minimize the number of animals used and their suffering.

### 4.3. Drugs

Human recombinant BDNF (Sigma, St. Louis, Missouri, USA) was diluted in sterile water and injected at a dose of 300 ng into the left lateral ventricle of mice (AP: −0.5 mm, L: −1.6 mm and DV: 2 mm; [59]). Before central drug administration, the animals were anesthetized for 20–30 s with diethyl ether. Sterile water was injected as a control (sham group). The volume of introcerebroventricularly administered solutions was 5mkl.

5-HT1A receptor agonist 8-OH-DPAT (8-hydroxy-2-(di-n-propylamino)tetralin, Research Biochemicals Inc., Wayland, Massachusetts, USA) was dissolved in saline and given intraperitoneally at a dose of 1.0 mg/kg for the estimation of 5-HT1A receptor functionality. The selectivity of 8-OH-DPAT for the 5-HT1A receptor was confirmed earlier by data that showed that blockading 5-HT7 receptors by the selective antagonist SB269970 had no effect on hypothermia evoked by the administration of 8-OH-DPAT [60].

5-HT2A receptor agonist DOI (1-(2,5-dimethoxy-4-iodophenyl)-2-aminopropane, Sigma, St. Louis, Missouri, USA) was dissolved in saline and administered intraperitoneally at a dose of 1 mg/kg to study the functionality of the 5-HT2A receptor.

### 4.4. Behavioral Assay

Behavioral testing was started 10 days after BDNF injection and carried out using the original EthoStudio software (Novosibirsk, Russia) [61]. EthoStudio software was applied for the automatic tracking of mouse behavior in the open-field and forced swim tests. In these tests we used transmitted (inverted) lighting for the automatic tracking of mouse behavior when the light was transmitted through the arena to the objective of a WEB camera, placed at 80 cm above the arena and connected to a PC computer via a USB 3.0 port. Since the mouse, regardless of its color (white, agouti or black), is opaque, it contrasts with the background in transmitted lighting [62].

### 4.5. Open-Field Test

A mouse was placed in a circle arena (40 cm in diameter) bordered with a white plastic wall (25 cm high) and illuminated through the mat and semitransparent platform with two halogen lamps of 12 W each, placed 40 cm under the platform. The mouse was placed near the wall and its movements were tracked for 5 min. The arena was carefully cleaned after each test. The horizontal locomotor activity (distance run, m), time spent in the center (%) and explored area (%) were measured automatically. The latter was estimated as the ratio of the area of the parts of the arena where the animal appeared (pixels associated with the mouse were detected) to the total area of the arena.

### 4.6. Forced Swim Test

Mice were placed in a clear cylindrical glass tank (30 cm height × 20 cm diameter) filled with water at a temperature of 25 °C for 2/3 of the volume. The water tank was placed on a semitransparent platform and brightly illuminated with two halogen lamps of 12 W each, placed 40 cm below the platform. Behavior was automatically evaluated for the last four minutes of the test using EthoStudio software by mobility level (rate of alteration of the animal silhouette). The last parameter is the rate of the number of animal-associated pixels that changed their position between two adjacent frames to the mean number of mouse-associated pixels in these frames. We used the mean rate calculated for the last four min of the test as the index of active resistance [62,63].

### 4.7. Intermale Aggression Test

Intermale aggression was assayed in the resident–intruder test as described early [20,64,65]. Briefly, a random-bred adult male albino mouse (intruder) was introduced into the home cage of the tested male (resident). Each intruder was used no more than two times. Behavior was tracked for 10 min with a digital camera (Sony, Tokyo, Japan), placed 80 cm above the cage. The intensity of aggression of the fighting mice was evaluated by the number of attacks toward the intruder and by the accumulating attacking time (s) during which the resident attacked the intruder. These behavioral indices were measured by an observer unaware of genotypes and treatments.

### 4.8. 5-HT1A Receptor Functional Response

The functional activity of the 5-HT1A receptor was estimated 15 days after BDNF injection by the quantification of the hypothermic response obtained after acute (i.p.) administration of 8-OH-DPAT (1 mg/kg) [66]. The body temperature was measured by means of a KJT thermocouple (Hanna Instruments, Singapore) with copper–constantan rectal probes for mice (Physitemp Instruments, Clifton, NJ, USA). For the estimation of hypothermic effect, two temperature values were analyzed: the first one was measured before and the second one was measured 20 min after acute 8-OH-DPAT administration to mice of the experimental and sham groups. 

### 4.9. 5-HT2A Receptor Functional Response

The functional activity of the 5-HT2A receptor was evaluated 17 days after BDNF injection by the number of 5-HT2A receptor-mediated head twitches evoked by the acute (i.p.) administration of DOI (1 mg/kg) [67]. The number of headshakes was measured over 20 min, starting 5 min after drug administration. 

### 4.10. High-Performance Liquid Chromatography (HPLC)

Three days after the last behavior test the animals were decapitated and the hypothalamus, nucleus accumbens, prefrontal cortex, hippocampus, striatum, subtantia nigra and midbrain raphe nuclei area were separated on ice. The tissue samples were frozen in liquid nitrogen and stored at −70 °C until the HPLC procedure. The brain structures were homogenized in 200 µL of 0.6 M HClO4 (Sigma–Aldrich, St. Louis, Missouri, USA). An aliquot of 180 µL of homogenate was centrifuged (15 min, 14,000× *g*, 4 °C) for protein precipitation. The supernatants were diluted two times with ultrapure water and the pellet was stored at −20 °C until protein quantitation by the Bradford method. Analysis of biogenic amine levels was performed by using an HPLC system containing the following components: an electrochemical detector (700 mV, DECADE IITM Electrochemical Detector; Antec, Zoeterwoude, The Netherlands), a glassy carbon flow cell (VT-03 cell 3 mm GC sb; Antec, The Netherlands), a CBM-20A system controller, a LC-20AD solvent delivery unit, an SIL-20A auto sampler and a DGU-20A5R degasser (Shimadzu Corporation, Columbia, Maryland, USA). The chromatographic separation of substances was carried out by isocratic elution at a flow rate of 1 mL/minute on a C18 column (5 mm particle size, L _ I.D. 100 _ 4.6 mm, Luna, Penomenex, Torrance, CA, USA) protected by a C8 security guard (Penomenex, Torrance, CA, USA) cartridge. The mobile phase was a mixture of 90% 50 mm phosphate buffer (Sigma Aldrich, St. Louis, Missouri, USA), containing 300 mg/L octanesulfonic acid sodium salt (Sigma Aldrich, St. Louis, Missouri, USA) (pH of 3.9), and 10% methanol (methanol (HPLC-grade), Fisher Chemical, Chicago, IL, USA). The temperature of the column was stabilized at 40 °C. Dopamine (DA), homovanillic acid (HVA), 3,4-dihydroxyphenylacetic acid (DOPAC), serotonin (5-HT) and 5-hydroxyindoleacetic acid (5-HIAA) (all supplied by Sigma-Aldrich, St. Louis, Missouri, USA) were used as standards for the calibration of the chromatographic system. The calibration curve of the standard mix (containing 0.5, 1 and 2 ng of each substance) was repeatedly assayed throughout the entire procedure. The areas of the peaks were estimated using LabSolutionLG/GC software version 5.54 (Shimadzu Corporation, Columbia, Maryland, USA) and calibrated against the corresponding standards. The contents of 5-HT, 5-HIAA, DA, HVA and DOPAC were expressed in ng/mg of protein, assayed by Bredford as described elsewhere [22]. The index of 5-HT and DA metabolism was calculated as the ratio of 5-HIAA/5-HT and DA/(HVA + DOPAC), respectively.

### 4.11. Statistical Analysis

All values were presented as means ± SEM and compared with two-way factorial ANOVA (factors “genotype” and “treatment” as well as their interaction) followed by Fisher’s post hoc analysis. The statistical significance was set at *p* < 0.05.

## Figures and Tables

**Table 1:** Two-way ANOVA results for the effects of factors “genotype” and “treatment” in addition to their interaction on serotonin (5-HT), its main metabolite, 5-hydroxyindoleacetic acid (5-HIAA), and the 5-HIAA/5-HT ratio in the different brain areas of B6-M76B and B6-M76C mice.

Structure	Two-Way ANOVA		
	Effect of Genotype	Effect of Treatment	Effect of Interaction
5-HT			
Hypothalamus	F1.29 < 1	F1.29 < 1	F1.29 < 1
N. accumbens	F1.29 < 1	F1.29 = 2.02, *p* > 0.05	F1.29 = 1.11, *p* > 0.05
Pref. cortex	F1.29 = 1.31, *p* > 0.05	F1.29 < 1	F1.29 < 1
Hippocampus	F1.29 = 1.22, *p* > 0.05	F1.29 < 1	F1.29 < 1
Striatum	F1.29 = 2.39, *p* > 0.05	F1.29 < 1	F1.29 = 1.25, *p* >0.05
S. nigra	F1.29 < 1	F1.29 = 2.03, *p* > 0.05	F1.29 < 1
Midbrain	F1.28 = 1.87, *p* > 0.05	F1.28 = 4.59 *	F1.28 = 4.97 *
5-HIAA			
Hypothalamus	F1.29 < 1	F1.29 < 1	F1.29 < 1
N. accumbens	F1.29 < 1	F1.29 = 1.41, *p* > 0.05	F1.29 < 1
Pref. cortex	F1.29 < 1	F1.29 < 1	F1.29 = 1.23, *p* > 0.05
Hippocampus	F1.29 < 1	F1.29 < 1	F1.29 < 1
Striatum	F1.29 < 1	F1.29 < 1	F1.29 < 1
S. nigra	F1.29 = 7.17 *	F1.29 < 1	F1.29 < 1
Midbrain	F1.28 < 1	F1.28 = 6.86 *	F1.28 = 3.82, *p* = 0.06
5-HIAA/5-HT			
Hypothalamus	F1.29 < 1	F1.29 < 1	F1.29 < 1
N. accumbens	F1.29 < 1	F1.29 < 1	F1.29 < 1
Pref. cortex	F1.29 < 1	F1.29 < 1	F1.29 < 1
Hippocampus	F1.29 < 1	F1.29 = 1.04, *p* > 0.05	F1.29 < 1
Striatum	F1.29 < 1	F1.29 < 1	F1.29 < 1
S. nigra	F1.29 = 6.63 *	F1.29 = 1.22, *p* > 0.05	F1.29 < 1
Midbrain	F1.28 = 10.31 **	F1.28 < 1	F1.29 = 1.18, *p* > 0.05

Genotype: B6-M76B or B6-M76C. Treatment: sham or BDNF. Interaction: genotype × treatment. *, *p* < 0.05; **, *p* < 0.01.

**Table 2:** Two-way ANOVA results for the effects of factors “genotype” and “treatment” as well as their interaction on dopamine (DA), its metabolites, 3,4-dihydroxyphenylacetic acid (DOPAC) and homovanillic acid (HVA), and the DOPAC+HVA/DA ratio in the different brain areas of B6-M76B and B6-M76C mice.

Structure	Two-Way ANOVA		
	Effect of Genotype	Effect of Treatment	Effect of Interaction
DA			
Hypothalamus	F1.29 < 1	F1.29 < 1	F1.29 < 1
N. accumbens	F1.28 < 1	F1.28 < 1	F1.28 < 1
Pref. cortex	F1.29 = 1.63, *p* > 0.05	F1.29 < 1	F1.29 = 1.61, *p* > 0.05
Striatum	F1.28 = 3.27, *p* = 0.08	F1.28 = 1.61, *p* > 0.05	F1.28 = 4.61 *
S. nigra	F1.29 = 3.49, *p* = 0.07	F1.29 = 2.02, *p* > 0.05	F1.29 < 1
Midbrain	F1.28 < 1	F1.28 = 4.72 *	F1.28 = 1.14, *p* > 0.05
DOPAC			
Hypothalamus	F1.29 < 1	F1.29 < 1	F1.29 = 5.66 *
N. accumbens	F1.28 = 1.78, *p* > 0.05	F1.28 < 1	F1.28 < 1
Pref. cortex	F1.29 < 1	F1.29 < 1	F1.29 < 1
Striatum	F1.28 = 8.89 **	F1.28 = 1.91, *p* > 0.05	F1.28 = 8.05 **
S. nigra	F1.29 < 1	F1.29 < 1	F1.29 = 1.22, *p* > 0.05
Midbrain	F1.28 < 1	F1.28 = 5.01 *	F1.28 < 1
HVA			
Hypothalamus	F1.29 < 1	F1.29 < 1	F1.29 < 1
N. accumbens	F1.28 <1	F1.28 < 1	F1.28 < 1
Pref. cortex	F1.29 < 1	F1.29 < 1	F1.29 = 2.05, *p* > 0.05
Striatum	F1.28 = 11.94 **	F1.28 = 4.93 *	F1.28 = 7.93 **
S. nigra	F1.29 = 9.85 **	F1.29 < 1	F1.29 < 1
Midbrain	F1.28 = 1.15, *p* > 0.05	F1.28 = 6.52 *	F1.28 < 1
DOPAC + HVA/DA			
Hypothalamus	F1.29 < 1	F1.29 = 1.32, *p* > 0.05	F1.29 = 2.87, *p* > 0.05
N. accumbens	F1.28 < 1	F1.28 < 1	F1.28 < 1
Pref. cortex	F1.29 = 1.74, *p* > 0.05	F1.29 < 1	F1.29 < 1
Striatum	F1.28 = 5.20 *	F1.28 < 1	F1.28 < 1
S. nigra	F1.29 < 1	F1.29 < 1	F1.29 < 1
Midbrain	F1.28 < 1	F1.28 = 1.22, *p* > 0.05	F1.28 = 1.22, *p* > 0.05

Genotype: B6-M76B or B6-M76C. Treatment: sham or BDNF. Interaction: genotype × treatment. *, *p* < 0.05; **, *p* < 0.01.

## Data Availability

The datasets generated during the current study are available from the corresponding author on reasonable request.
